# In Vitro Activity of Amphotericin B in Combination with Statins against Clinical and Environmental *Rhizopus oryzae* Strains

**Published:** 2019-05

**Authors:** Mahsa NAEIMI ESHKALETI, Parivash KORDBACHEH, Seyed Jamal HASHEMI, Mehraban FALAHATI, Farideh ZAINI, Hossein MIRHENDI, Mahin SAFARA, Leila HOSSEINPOOR

**Affiliations:** 1. Department of Medical Parasitology and Mycology, School of Public Health, Tehran University of Medical Sciences, Tehran, Iran; 2. Department of Medical Parasitology and Mycology, School of Medicine, Iran University of Medical Sciences, Tehran, Iran

**Keywords:** Mucormycosis, Mucorales, Amphotericin B, Atorvastatin, Lovastatin, *Rhizopus oryzae*

## Abstract

**Background:**

Mucormycosis is an acute and invasive fungal infection with a high mortality rate. Mucorales are less sensitive than other types of fungi to most antifungal agents. Amphotericin B (AMB) is one treatment option for this infection, but in recent studies, the antifungal activity of statins against Mucorales was shown. Therefore, therapy that combines AMB with these agents may have better effects in management of patients with mucormycosis. We evaluated the in vitro activity of AMB alone and in combination with statins, against Mucorales.

**Methods:**

Susceptibility profiles of AMB alone and in combination with two statins, atorvastatin (ATO) and lovastatin (LOV) determined against clinical (n: 15) and environmental (n: 5) *Rhizopus oryzae* isolates, obtained between Jan 2009 and Oct 2016 from patients with uncontrolled diabetes mellitus and cancer referred to the Department of Parasitology and Medical Mycology of Tehran University of Medical Sciences, Tehran, Iran. It was performed by microdilution method, based on the Clinical and Laboratory Standard Institute (CLSI) M38-A2 guideline.

**Results:**

All clinical and environmental isolates were susceptible to AMB (MIC≤1 μg/mL). The results of the interactions between AMB and the two statins were positive. The AMB-ATO (GM: 0.13 μg/Ml) combination produced greater activity than the AMB-LOV (GM: 0.26 μg/mL) combination. AMB, in combination with ATO and LOV, reacts positively against clinical and environmental *R. oryzae* isolates.

**Conclusion:**

This combination strategy may lead to more effective treatment of mucormycosis and fewer side effects using low dose of AMB.

## Introduction

Mucormycosis is a serious and life-threating fungal disease caused by fungi belonging to the order Mucorales. The most common etiologic agents are *Rhizopus* spp., whereas the *Mucor*, *Rhizomucor*, *Absidia*, *Saksenaea*, *Apophysomyces* and *Cunninghamella* species make up a smaller percentage of clinical isolates (1–3).

The most common risk factors for this infection include uncontrolled diabetes mellitus, solid organ and bone marrow transplantation, hematological malignancies, extensive burn and trauma, corticosteroids and deferoxamine therapy (1, 4).

Mucorales enter the body through inhalation of spores or permeation of them into damaged skin or mucosa, causing acute inflammatory reactions (5). Mucormycosis is the third most common opportunistic fungal invasive infection after candidiasis and aspergillosis (6, 7).

Distinguishing between mucormycosis and aspergillosis usually is difficult, and Mucorales are resistant to most antifungal agents that are active against causative agents of invasive aspergillosis (1, 8).

Given the growing number of patients with predisposing factors and the occurrence of antibiotic-resistant strains, the incidence of mucormycosis has increased dramatically over the last several years (9). Treating mucormycosis is challenging, and outcomes typically are unfavorable because, in most cases, the causative agents of this infection have inherent resistance to a large number of antifungal drugs (10, 11). Besides amphotericin B (AMB) that is the top choice for treating mucormycosis, posaconazole also demonstrates appropriate activity against the causative agents of this infection (11, 12). However, despite these treatments, the overall outcome of mucormycosis remains poor, with a high mortality rate. Therefore, the development of novel strategies to treat this infection are necessary. Statins are a class of drug used mainly to lower cholesterol in humans. However, in recent studies, the in vitro antifungal activity of these agents was shown to work against filamentous and yeast fungi (13–15).

Vast using of statins has been hypothesized that could protect, partially, in patients suffering from diabetes against Mucormycosis (6, 16, 17). They can be used in combination with conventional antifungal drugs in order to achieve a therapy that requires lower doses and less toxicity (18, 19) such as AMB that is toxic and has side effects (20). Therefore, combining AMB with other compounds that have antifungal effects can improve the efficacy of the treatment in lower concentrations (21).

Effects of statins on *Mucorales* strains have not been studied in Iran also the aim of this study was to evaluate the effects of AMB in combination with two statins against clinical and environmental *Mucorales* strains identified by sequencing internal transcribed spacers.

## Materials and Methods

### Strains

Twenty clinical (n=15) and environmental (n=5) isolates of *Mucorales* species were studied. The clinical isolates were obtained between Jan 2009 and Oct 2016 from patients with uncontrolled diabetes mellitus and cancer referred to the Department of Parasitology and Medical Mycology of Tehran University of Medical Sciences. During this survey, 5 environmental *Rhizopus* sp*.* were collected from laboratory spaces also studied. Then, all strains were identified to the species level by analyzing the internal transcribed spacer (ITS) regions using PCR product sequencing (22). All isolates were cultured onto potato dextrose agar (micro media) at 35 °C for 5 d.

### Antifungal susceptibility testing

**Statins and antifungal agent:** Amphotericin B (AMB, Sigma-Aldrich) was purchased as a standard powder and dissolved in dimethylsulphoxide (DMSO). The statins used in this study were lovastatin (LOV Mevacor, Poursina Company) and atorvastatin (ATO Atorvax, Tehran Chemistry Company), provided as a standard powder and prepared in methanol at a concentration of 256 mg each in 50ml methanol. Stock solutions of these drugs were stored at −70 °C until use.

**In vitro antifungal susceptibility testing:** The in vitro antifungal susceptibility was determined using Clinical and Laboratory Standard Institute (CLSI) document (M38-A2) (23). Briefly, AMB and the two statins were diluted in RPMI 1640 medium (Sigma-Aldrich, St. Louis, MO, USA) with L-glutamine, without bicarbonate, buffered to pH 7 with 0.165 mol L-1 morpholinepropanesulfonic acid (MOPS, Sigma-Aldrich, St. Louis, MO, USA). The final concentration of AMB, LOV and ATO ranged from 0.016 to 16. Fungal strains were cultured onto potato dextrose agar (PDA) slants for 5–7 d at 35 °C. Spore suspensions were prepared by washing the surface of the slants with sterile saline containing 0.05% Tween 80; large fragments were allowed to settle. The final inocula were 0.5–5×10^4^ conidia/mL in RPMI-1640 buffered with MOPS. The statins-AMB combinations were examined using the chequerboard broth microdilution method using two-fold dilutions from each drug. First, 50μl of AMB and 50μl of each statin were placed in the well, and then 100μl of sporangiospore suspension was added to microtiter plates and incubated at 35 °C for 24 or 48 h. The minimum inhibitory concentration (MIC) of AMB, LOV and ATO were determined visually using a mirror to compare the growth in wells with drugs and in control wells without drugs. *Candida krusei* (ATCC 6258) and *C. parapsilosis* (ATCC 22019) were used as quality control. The geometric means (GM) and ranges of the MIC were determined for each drug alone and in combination after 24h. If no growth was observed, the incubation time was extended to 48h. In addition, the MIC50 (minimal concentration that inhibits 50% of isolates) and MIC90 (minimal concentration that inhibits 90% of isolates) were calculated for all drugs using the aforementioned MIC determination criteria. All experiments were performed two times for each strain.

## Results

Twenty clinical and environmental strains were identified as *R. oryzae* by PCR sequencing of ITS regions. The MIC data of AMB and the two statins are summarized in Table 1.

**Table 1:** MIC results of AMB/ATO, LOV combination against 20 strains of *R. oryzae*

***Strain***	***Source***	***MIC of LOV (μg/m)***	***MIC of ATO (μg/m)***	***MIC of AMB alone (μg/m)***	***MIC of AMB in combination with LOV (μg/m)***	***MIC of AMB in combination with ATO (μg/m)***
*R. oryzae*	Clinical-1	>16	4	0.5	0.5	0.25
*R. oryzae*	Clinical-4	>16	8	0.5	0.5	0.25
*R. oryzae*	Clinical-7	>16	16	0.5	0.25	0.031
*R. oryzae*	Clinical-8	>16	8	0.063	0.063	0.063
*R. oryzae*	Clinical-9	>16	2	0.031	0.031	0.031
*R. oryzae*	Clinical-10	>16	8	0.5	0.5	0.25
*R. oryzae*	Clinical-11	>16	8	0.5	0.25	0.25
*R. oryzae*	Clinical-12	>16	8	1	0.5	0.25
*R. oryzae*	Clinical-13	>16	8	0.25	0.25	0.25
*R. oryzae*	Clinical-14	>16	16	0.5	0.25	0.063
*R. oryzae*	Clinical-15	>16	8	1	0.5	0.063
*R. oryzae*	Clinical-16	>16	16	1	1	1
*R. oryzae*	Clinical-17	>16	16	0.5	0.5	0.031
*R. oryzae*	Clinical-19	>16	>16	0.5	0.25	0.25
*R. oryzae*	Clinical-20	>16	>16	0.5	0.25	0.25
*R. oryzae*	Environmental-2	>16	8	1	0.25	0.25
*R. oryzae*	Environmental-3	>16	8	1	0.25	0.063
*R. oryzae*	Environmental-5	>16	8	0.5	0.25	0.25
*R. oryzae*	Environmental-6	>16	>16	0.5	0.25	0.063
*R. oryzae*	Environmental-18	>16	>16	0.063	0.063	0.063
MIC range			2–>16	0.031–1	0.031–1	0.031–1
MIC50			8	0.5	0.25	0.25
MIC90			>16	1	0.5	0.25
GM			9.5	0.38	0.26	0.13

All clinical and environmental isolates were susceptible to AMB (MIC≤1); the MIC range for AMB was 0.031 to 1 μg/mL, GM MIC; 0.38 μg/mL, MIC50; 0.5 μg/mL, MIC90; 1 μg/mL. Between the two statins, ATO (GM0: 9.5 μg/mL) exhibited better activity against *R. oryzae* isolates than LOV (MIC>16). The results of the interactions between AMB and the two statins (LOV and ATO) were positive. The AMB-ATO (GM: 0.13 μg/Ml) combination exhibited more activity than the AMB-LOV (GM: 0.26 μg/mL) combination. When AMB was combined with ATO, the MIC of 15 isolates of *R. oryzae* were reduced. The MIC50 and MIC90 of the AMB-ATO combination were 0.25 μg/mL, which was less than with AMB alone.

## Discussion

Mucormycosis is an invasive, opportunistic mold infection that affects patients with impaired immune systems. The causative agents of this infection belong to the order Mucorales, genus *Rhizopus,* and are by far the most common strains recovered from patients (6). Early diagnosis and appropriate treatment are critical for better prognosis in patients with mucormycosis (24). Many previous studies reported antifungal activity of statins against yeasts and molds (13, 25) and in the present study, 15 clinical and 5 environmental isolates of *R. oryzea* were evaluated for antifungal susceptibility testing to AMB alone and in combination with two statins, ATO and LOV. Although amphotericin B is usually the choice for treating mucormycosis, the gold standard therapy for this infection has not been found yet (8). The mortality rate remains high, even with continuous therapy with this treatment strategy (1). Therefore, there is a serious need to develop antifungal therapeutic strategies against mucormycosis that have less severe side effects.

The results of our study showed that AMB in combination with ATO (GM: 0.13 μg/mL) exhibited more activity against the isolates than the AMB-LOV combination (GM: 0.26 μg/mL) or AMB alone (GM: 0.38 μg/mL). AMB is toxic and has side effects (20); therefore, the ability to use the drug in lower concentrations by combining it with other effective agents, such as statins, would be beneficial for a less toxic therapy. One isolate of *R.oryzea* was evaluated after treatment with the AMB-ATO combination. The MIC of AMB and ATO was reduced, and this combination produced an additive effect on the inhibition of fungi growth (26).

Statins in high concentrations are active against some clinically important fungi (27–29). In our study, when LOV and ATO were evaluated alone against *R. oryzea* isolates, the MIC data were high (GM LOV: >16 μg/mL and GM ATO: 9.5 μg/mL). Statins in these concentrations are not appropriate for use alone as antifungal drugs to treat invasive fungal infections because these MICs are higher than the maximum available concentration ranges in human serum. Therefore, in treating invasive fungal infections, the application of statins is only feasible in combination with other antifungal agents (30).

Statins can be divided into two groups: 1) natural statins, which are metabolites of microorganisms, such as LOV, and 2) completely synthetic compounds, such as ATO (31). Synthetic statins are more effective against pathogenic fungi than natural statins (18, 32). Likewise, in the present study, the MIC data of ATO, a synthetic statin, indicated that it was more effective than LOV, both alone and in combination with AMB.

## Conclusion

AMB in combination with ATO and LOV showed a positive activity against the clinical and environmental *R. oryzae* isolates. This combination strategy may help clinicians treat mucormycosis in lower drug concentrations and without serious side effects.

## Ethical considerations

Ethical issues (Including plagiarism, informed consent, misconduct, data fabrication and/or falsification, double publication and/or submission, redundancy, etc.) have been completely observed by the authors.
